# Multi-cavity Primary Effusion Lymphoma in a Cirrhotic Patient: A Rare Cause of Chylous Effusion

**DOI:** 10.7759/cureus.109242

**Published:** 2026-05-19

**Authors:** Trilok Chand, Seema Rab, Asiya Pathan, Hiran Ravindran

**Affiliations:** 1 Pulmonology Department, Burjeel Hospital, Abu Dhabi, ARE; 2 In-Patient Department, Burjeel Hospital, Abu Dhabi, ARE; 3 Histopathology, Burjeel Medical City, Abu Dhabi, ARE

**Keywords:** chylothorax, chylous ascites fluid overload-associated large b-cell lymphoma, liver cirrhosis, primary effusion lymphoma, refractory pleural effusion

## Abstract

Primary effusion lymphoma (PE) is a rare form of non-Hodgkin lymphoma characterized by malignant lymphatic effusion in the pleural cavity. Non-traumatic chylothorax is usually caused by lymphomas with a discrete lymph node mass. Our report presents a case where chylothorax and chylous ascites were clinical presentation of primary effusion lymphoma (PEL), a rare entity that does not show the typical presence of an identifiable malignant mass. Managing PEL complicated by chylothorax is generally more challenging and has a poor prognosis, as illustrated by a patient in this case who died weeks after diagnosis.

## Introduction

Primary effusion lymphoma (PEL) stands as an uncommon aggressive B-cell non-Hodgkin lymphoma which primarily affects immunocompromised patients, especially those infected with human immunodeficiency virus (HIV) [1]. The disease appears rarely in patients with liver cirrhosis, which creates multiple challenges for diagnosis and management.

Medical literature lacks precise data about PEL frequency in cirrhotic patients, although it has appeared among elderly individuals with liver cirrhosis [2].

PEL is defined by effusions in body cavities that can be associated with lymphomas without detectable tumours or lymph node masses. The occurrence of PEL in our patient without overt immunosuppression, as indicated by negative serologies for HIV, human herpesvirus-8 (HHV-8), and other known predisposing infections, challenges the conventional understanding of PEL pathogenesis.

Chylothorax is an uncommon presentation of PEL, and it may complicate diagnosis further. This case report describes the rare case of chylothorax with PEL in a patient with cirrhosis, underscoring the need for a higher index of suspicion for atypical presentations for accurate and appropriate diagnosis and management.

## Case presentation

An Asian lady aged 60 years, known to have a past medical history of hypothyroidism, non-alcoholic liver cirrhosis and recurrent bilateral pleural effusion, presented with increased dyspnoea and generalised weakness for over three days.

**Figure 1:**
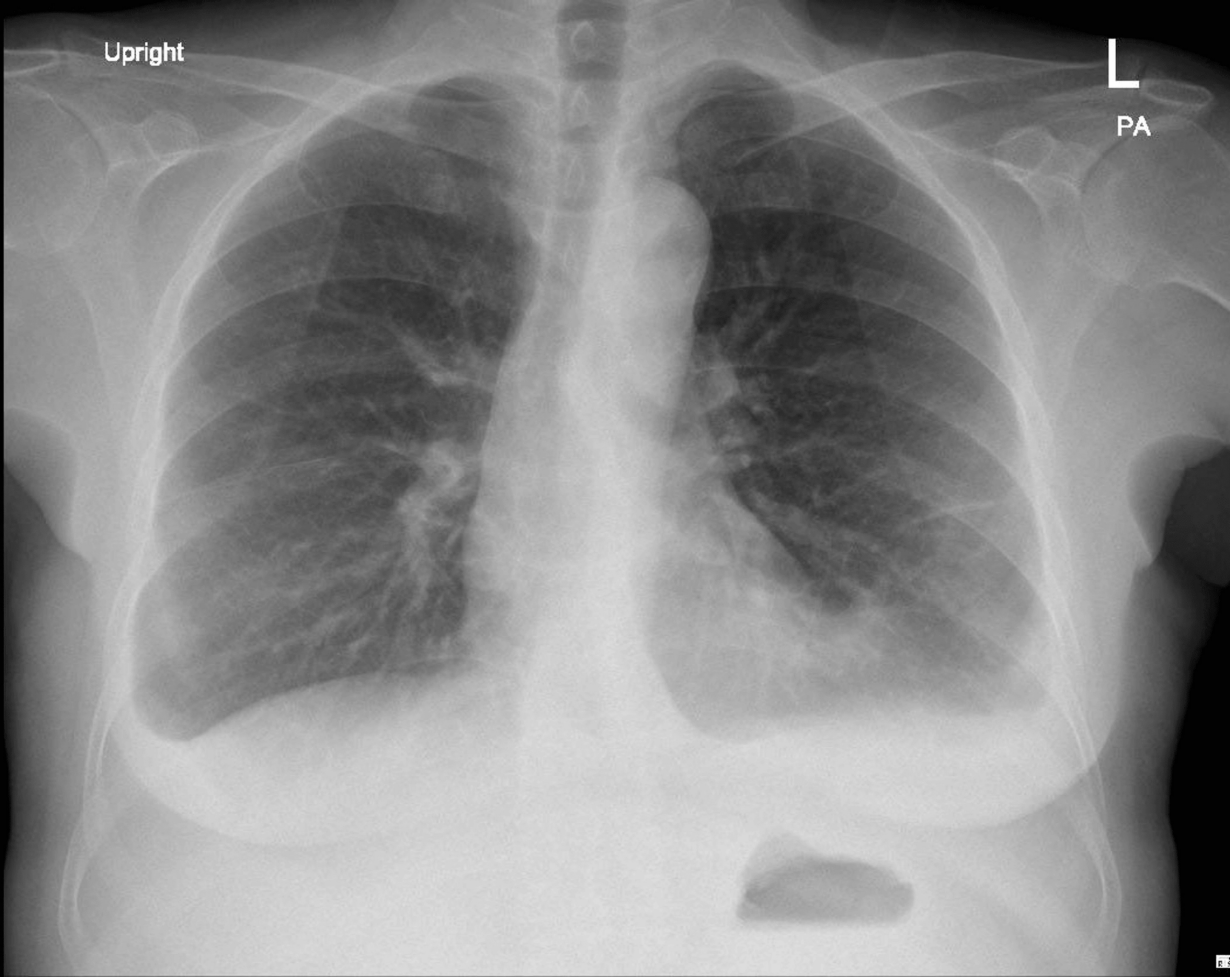
Chest X-ray showing blunting of bilateral costophrenic angles-mild pleural effusion.

During admission, the patient had a stable condition of vital signs except for mild tachypnoea. Physical chest examination showed decreased breath sounds, noted at the lung bases bilaterally. Chest X-ray showed bilateral costophrenic angle blunting, consistent with pleural effusion (Figure 1).

Further lab findings revealed elevated white blood cell counts with lymphopenia, thrombocytosis, and high D-dimer levels. Hypoalbuminemia, hyperkalemia, elevated creatinine and urea, and raised levels of aspartate aminotransferase were also documented. HIV status, hepatitis B surface antigen and hepatitis C virus (HCV) antibody were negative (Table 1).

**Table 1:** Result of blood investigations. CRP: C-reactive protein, ALT: alanine aminotransferase, AST: aspartate aminotransferase, HCV: hepatitis C virus, ESR: erythrocyte sedimentation rate

Parameters	Patient values	Reference range
Hemoglobin	14.40 gm/dl	12-15 gm/dl
WBC	11.48x10^3 /mcL	4-10.5x10^3 /mcL
Platelet counts	963x10^9 /L	150-410x10^9 /L
CRP	5.8 mg/L	0-5 mg/L
D-dimer	10.21 mcFEU/ml	0-0.5 mcFEU/ml
Serum Albumin	22 g/L	39.7-49.4 g/L
Serum Protein	43 g/L	64-83 g/L
Serum Potassium	6.6 mmol/L	3.5-5.1 mmol/L
Urea	8.26 mmol/L	3.5-7.2 mmol/L
Creatinine	83 mcmol/L	44-80 mcmol/L
ALT	14 U/L	0-33 U/L
AST	85 U/L	0-32 U/L
HBs antigen	Negative	Negative
HCV antibody	Negative	Negative
β2-microglobulin	>20000 ng/ml	609.0-2366.0 ng/ml
Kappa level	46.28 mg/L	3.30-19.40 mg/L
Lambda level	35.84 mg/L	5.71-26.30 mg/L
Quantiferon TB GOLD	Negative	Negative
ESR	15	0-25 mm at 1 hour

**Figure 2:**
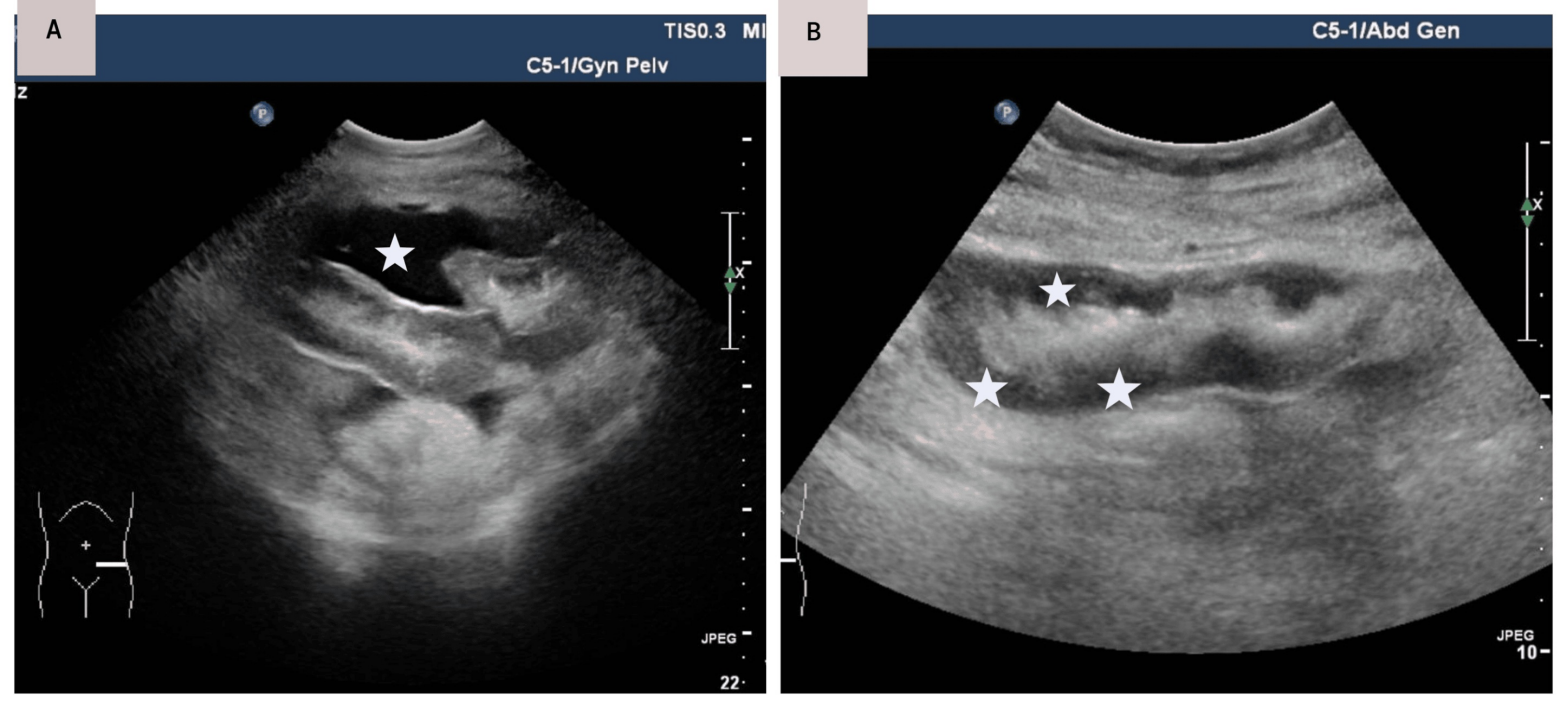
Ultrasound (USG) of the abdomen and pelvis (white asterisk marks) demonstrating ascites (A) and bowel wall thickening (B).

The abdominal ultrasound demonstrated mild to moderate ascites associated with thickening of the bowel wall, the findings suggesting fluid overload by cirrhosis and renal dysfunction (Figure 2). Antibiotics (amoxiclav), low-dose diuretics, and supportive therapy were initiated.

**Figure 3:**
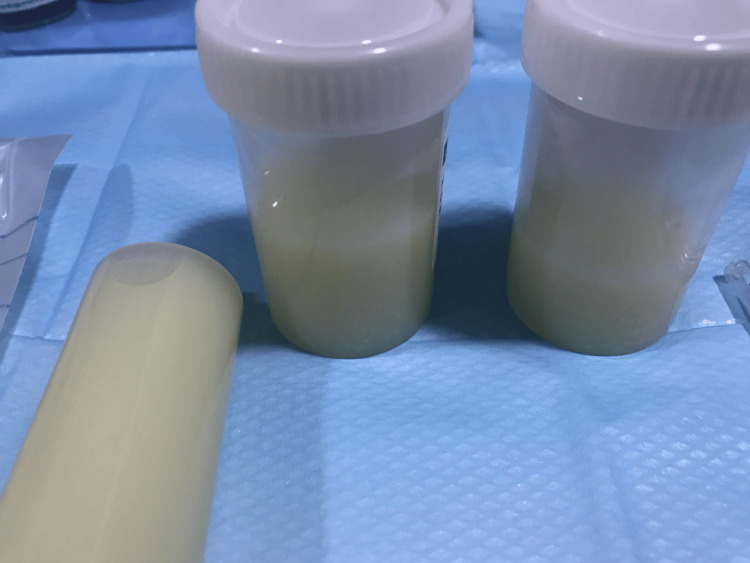
Sample bottles containing milky colour pleural fluid.

On ultrasound-guided thoracentesis, a milky-appearing fluid was noted (Figure 3). The analysis of pleural fluid showed high levels of lactate dehydrogenase (LDH), low glucose, and high protein, indicative of an exudative nature. Elevated triglyceride levels (4.25 mmol/L) with a normal amount of cholesterol (0.96 mmol/L) in pleural fluid were indicative of a chylous effusion (Table 2).

**Table 2:** Results of pleural fluid analysis. LDH: lactate dehydrogenase, ADA: adenosine deaminase

Pleural fluid parameters	Patient values	Reference range
Protein	23.1 g/L	10-20 g/L
Glucose	1.48 mmol/L	4.44-7.77 mmol/L
LDH	4370 U/L	<200 U/L-Transudative, >200 U/L- Exudative
Triglyceride	4.25 mmol/L	<0.56 mmol/L
Cholesterol	0.96 mmol/L	<5.18 mmol/L for Chylothorax
ADA	164.40 U/L	<40 U/L
Lymphocytes	100% and atypical	<15% and typical

Significantly high pleural fluid adenosine deaminase (ADA; 164.40 U/L) was more in favour of a lymphoproliferative disorder than tuberculosis. Pleural fluid ADA is often very high in lymphoma because ADA is essential for DNA metabolism and is highly concentrated in lymphocytes, and T-cell and B-cell proliferation and cell turnover in lymphoma is quite high compared with tuberculosis. She was kept nil per oral and initiated on total parenteral nutrition and subcutaneous octreotide.

**Figure 4:**
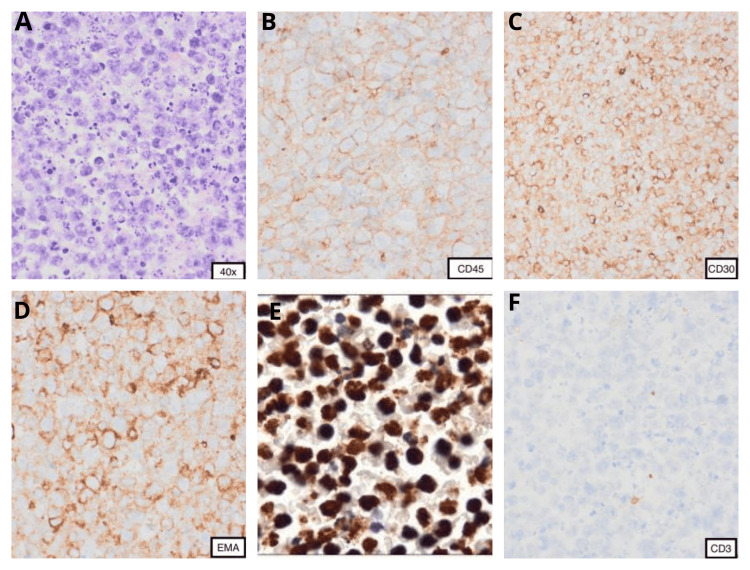
Histopathology slides showing atypical lymphoid cells on H&E high power showing plasmacytoid and immunoblastic morphology (Slide A). Immunohistochemistry high power showing immunopositivity for CD45 (Slide B), CD30 (Slide C), EMA (Slide D) and HHV-8 (antibody to LANA-1, Slide E). Rare cells were positive for CD3 (Slide F). H&E: hematoxylin and eosin, HHV-8: human herpesvirus-8

Pleural fluid cytology showed the presence of atypical lymphoid cells with plasmacytoid and immunoblastoid morphology. Some of the atypical lymphoid cells had enlarged nuclei, prominent nucleoli, and karyorrhectic debris. Intermixed with these cells were neutrophils and mesothelial cells. Immunohistochemistry showed positivity for CD45, CD30, EMA, MUM-1, lambda, and kappa (almost kappa-restricted), but negativity for CD20, CD79a, p53, CD138, PAX5, ALK, and pancytokeratin (CKAE1/AE3). Rare aberrant CD3-positive cells are also noted (Figure 4A-4F).

Given the absence of lymphadenopathy and the presence of atypical lymphoid cells in pleural fluid, the differential diagnosis on pathology is primary effusion lymphoma, fluid-overload-associated large B-cell lymphoma (FO-LBCL), and anaplastic large cell lymphoma, ALK-negative. FO-LBCL usually shows a complete B-cell phenotype (CD20, CD79a, CD19). Contrastingly, PEL is proliferation of mature B-cells lacking the pan-B-cell markers, frequently expressing terminal differentiation markers (HLA-DR, CD30, EMA, CD38, VS38c, CD138, IRF4/MUM-1, and Blimp1). Expression of MUM-1/IRF4 is a distinguishing trait of PEL among lymphomas involving serous body cavities. ALK-negative anaplastic large cell lymphomas usually demonstrate T-cell positivity. In this case, clinical presentation and immunoprofile (CD45-positive, CD20-negative, CD79a-negative, PAX5-negative, CD3-negative, with EMA-positive, CD30-positive and MUM1-positive) supported a diagnosis of PEL. HHV-8 LANA-1 nuclear positivity in the atypical pleural fluid cells, together with this immunophenotype and presentation, is highly supportive of Primary Effusion Lymphoma related to liver cirrhosis.

In their study, Carbone et al. demonstrated that MUM-1/IRF4 is expressed in 100% of the cases examined, indicating that it is probably involved in the pathogenesis of the disease. Its expression favours the view that PEL arises from a post-germinal centre stage of differentiation and may assist in differential diagnosis and possible viral immune evasion mechanisms [3].

Further blood tests revealed markedly raised values of β2-microglobulin (>20,000 ng/ml). Serum protein electrophoresis showed reduced total protein, albumin, β1-β2, and gamma globulin, while the alpha fractions were normal. Analysis of serum free light chains disclosed elevated kappa levels (46.28 mg/L) and lambda levels (35.84 mg/L), while the ratio remained 1.29, which is normal, thus confirming the presence of elevated polyclonal immunoglobulin free light chains (FLCs). An increase in both monoclonal and polyclonal free light chains is indicative of poor prognosis in B-cell lymphomas [4,5].

**Figure 5:**
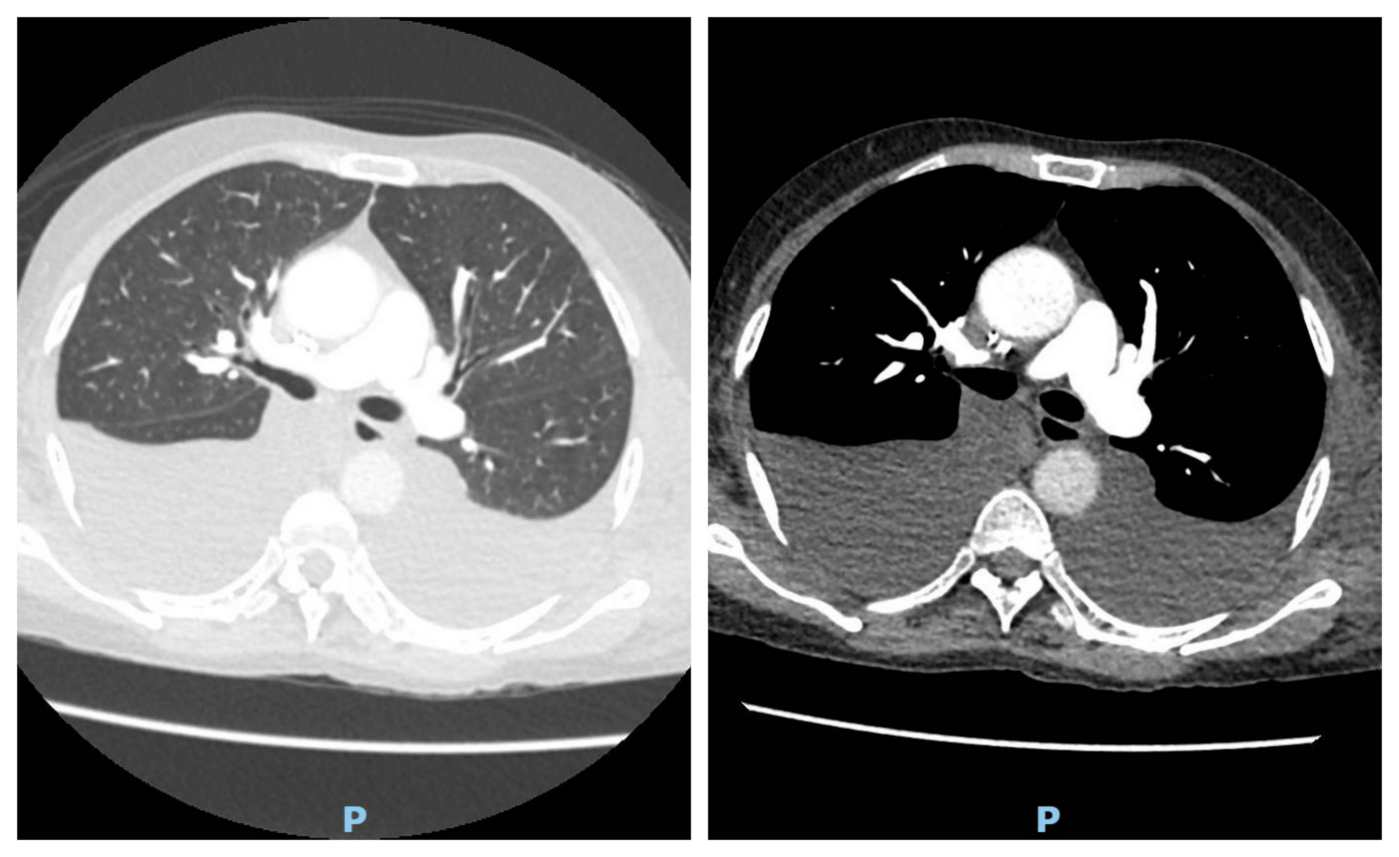
CT chest (lung and mediastinal windows) showing bilateral pleural effusion.

The CT pulmonary angiogram ruled out pulmonary embolism and lymphadenopathy but showed bilateral pleural effusion, basal lung collapse, fibro-bronchiectasis, and a calcified granuloma (Figure 5). The CT of the abdomen and pelvis demonstrated signs of generalised anasarca, cirrhosis, massive ascites, and thickening of the peritoneum.

Bilateral chest tubes and a peritoneal catheter were inserted. Ascitic fluid, although dark straw, was also consistent with a chylous lymphomatous effusion. The patient became continuously worse with metabolic acidosis, hypotension, and hyperlactataemia and required ICU admission. Repeat investigations indicated worsening anaemia, high WBC count, elevated INR, and persistent hypoalbuminaemia. Her model for end-stage liver disease-sodium (MELD-Na) score was 22, and Child-Pugh class C (score 10) indicated advanced decompensated cirrhosis and high risk of mortality. Intravenous fluids, bicarbonate, noradrenaline, and meropenem were administered.

Regardless of the ICU monitoring, she was still reliant on a small amount of vasopressors, required oxygen therapy, and was intermittently given diuretics for her oliguria. Thoracoscopy or video-assisted thoracoscopic surgery (VATS) biopsy was delayed due to her poor performance status (Eastern Cooperative Oncology Group {ECOG} performance status 4 and Karnofsky Performance Status {KPS} score 30), and chemotherapy was not initiated. The family then transferred her to another facility, and she died after 10 days.

## Discussion

Among the elderly or those who are immunocompromised, the incidence of PEL is higher and it contributes about 4% to HIV-related non-Hodgkin lymphomas [6,7] and about 1% to lymphoma cases without HIV-related causes [8]. The ratio is strongly in favor of males, at 6 to 1 [9]. Other causes where PEL is triggered are HHV-8 infections in immunocompromised patients, post-transplant immunosuppression, and cirrhosis.

There is a correlation between chronic volume overload syndromes, such as those seen in end-stage renal disease and cirrhosis, and PEL [10]. Chronic serosal irritation from persistent fluid overload may aid in promoting malignant transformation [11].

The WHO 5th edition has recently put forth the term "fluid overload-associated large B-cell lymphoma (FOALBCL)" to denote these cases, which look a lot like PEL but are negative for HIV and HHV-8 [12].

Chylothorax is an uncommon complication, characterised by triglycerides studied above 110 mg/dL (~1.24 mmol/L), cholesterol less than 200 mg/dL (~5.18 mmol/L), and further confirmed by appearance of chylomicrons on lipoprotein electrophoresis when values are borderline [13-16]. For more than 50% of instances of non-traumatic chylothorax, malignancy is responsible, usually considering lymphoproliferative disorders [17]. PEL without masses or lymphadenopathy is an uncommon cause.

Breathlessness, cough, chest pain, fever, night sweats, and weight loss [18,19] are common symptoms of malnutrition and electrolyte loss, which occur commonly due to continuous protein, fat, and vitamin depletion [14].

Diagnosis is mainly through effusion cytology and immunophenotyping, which are, in most cases, positive for HHV-8 and negative for pan-B/T markers. Elevated serum B2-micoglobulin in the presence of a normal glomerular filtration rate suggest increased ß2M production or release. Increased levels may be seen in lymphoproliferative diseases such as multiple myeloma, ß- cell chronic lymphocytic leukaemia, Hodgkin's disease, non-Hodgkin's lymphoma, systemic lupus erythematosus, rheumatoid arthritis, Sjogren's syndrome, Crohn's disease, and certain viral infections, including cytomegalovirus, non-A and non-B hepatitis and infectious mononucleosis. Early diagnosis is crucial because of the rapid progression and the resulting poor prognosis.

Management entails a pulmonology, oncology, pathology, and surgery team. Treatments include cyclophosphamide, doxorubicin, vincristine, and prednisone (CHOP); etoposide, prednisone, vincristine, cyclophosphamide, and doxorubicin (EPOCH); cyclophosphamide, doxorubicin, and etoposide (CDE) chemotherapy; pleurodesis; and supportive measures [20]. Poor response with median survival <6 months [20]. Prognosis worsens with multi-cavity involvement, from 18 months with a single cavity to four months with multiple [19].

With cirrhosis, multi-cavity effusion, poor performance status, and no chance for chemotherapy - all poor prognostic factors - our patient survived only weeks post-diagnosis.

## Conclusions

Rare yet highly aggressive clinical entities, lymphomas, and chylothoraces require early recognition and multidisciplinary treatment. Unless clinical vigilance is maintained, the prognosis remains poor, especially with the presence of cirrhosis and multi-cavity disease.
